# Lotus seed *(Nelumbinis semen)* extract: anticancer potential and chemoprofiling by *in vitro*, *in silico* and GC-MS studies

**DOI:** 10.3389/fchem.2024.1505272

**Published:** 2024-12-13

**Authors:** Vijaya Jyothi Mallela, Mithun Rudrapal, D. S. N. B. K. Prasanth, Praveen Kumar Pasala, Atul R. Bendale, Soumya Bhattacharya, Sahar M. Aldosari, Johra Khan

**Affiliations:** ^1^ Raghavendra Institute of Pharmaceutical Education and Research, Jawaharlal Nehru Technological University Anantapur, Anantapur, India; ^2^ Department of Pharmaceutical Sciences, School of Biotechnology and Pharmaceutical Sciences, Vignan’s Foundation for Science, Technology and Research, Guntur, India; ^3^ School of Pharmacy and Technology Management, SVKM’s Narsee Monjee Institute of Management Studies (NMIMS), Hyderabad, India; ^4^ Mahavir Institute of Pharmacy, Nashik, India; ^5^ Guru Nanak Institute of Pharmaceutical Science and Technology, Kolkata, India; ^6^ Department of Medical Laboratory Sciences, College of Applied Medical Laboratory Sciences, Majmaah University, Al Majma’ah, Saudi Arabia; ^7^ Health and Basic Science Research Center, Majmaah University, Al Majma’ah, Saudi Arabia

**Keywords:** lotus seeds, methanolic extract, GC-MS, chemoprofiling, anticancer, phytochemicals, molecular docking

## Abstract

Lotus seeds, also known as Nelumbinis semen, has been utilized for over 7,000 years as vegetable, functional food and medicine. In this study, we primarily investigated the anticancer effects of lotus seed extracts, particularly of the methanolic extract (MELS) on cell proliferation inhibition, apoptosis induction and cell cycle arrest in ovarian cancer cell lines. Further, we studied the phytochemical composition of the MELS by gas chromatography-mass spectrometry (GC-MS) analysis. Additionally, molecular docking was performed in order to substantiate the *in vitro* anticancer effect by *in silico* inhibitory study of human survivin protein. Our *in vitro* study demonstrated significant inhibition of SKOV3 (IC_50_: 79.73 ± 0.91), A2780 (IC_50_: 100.18 ± 2.42), SKOV3-CisR (IC_50_: 115.87 ± 2.2) and A2780-CisR (IC_50_: 138.86 ± 2.46) cells by MELS, compared to acetone, petroleum ether, n-hexane extracts, and the standard drug, cisplatin. Furthermore, MELS resulted in a substantial increase in apoptosis cell count to 78% in A2780-CisR cells and 82% in SKOV3-CisR cells, whereas a significant reduction in the G1 and G2/M phases of cells treated with MELS when compared to the control group. To identify the potential phytocompounds present in the MELS, we conducted GC-MS analysis, which led to the identification of 14 compounds. Molecular docking analysis revealed that oleic acid, stigmast-5-en-3-ol, phytol and glyceryl linolenate exhibited remarkable binding affinities of −6.1, −5.9, −5.8 and −5.6 kcal/mol, respectively against survivin. Our findings suggest that certain phytochemicals presented above found in MELS may have therapeutic potential for management of ovarian cancer.

## 1 Introduction

Ovarian cancer (OC) is the fourth most common cause of cancer-related death among women in the Western world (Kohn et al., 2003; Mills et al., 2003). In the year 2020, there were an estimated 21,750 newly diagnosed cases of ovarian cancer, accounting for around 1.2% of all cancer cases. The expected death toll is at 13,940. The incidence of ovarian cancer risk is 1 in 70 women. Annually, OC causes 150,000 fatalities worldwide, making it the most lethal among various gynaecological cancers. Chemotherapy, laser therapy, radiation, gene therapy and surgery are some of the interventions now being used or tested to disrupt the proliferation of cancer cells (Ruibin et al., 2017; Senapati et al., 2018). Most ovarian cancer patients experience a recurring and worsening condition, as they develop a resistance to different types of standard chemotherapy medicines (Grosso et al., 2013). Furthermore, a majority of synthetic drugs employed in cancer therapy exhibit substantial harm to healthy cells. Conversely, diverse naturally-occurring phytochemicals found in plants have exhibited specific toxicity towards certain types of human cancer cells, while causing minimal harm to normal cells (Devi et al., 2015).

Survivin is a member of the inhibitor of apoptosis (IAP) protein family and plays a significant role in controlling the mitotic process and defending against apoptosis inhibition, which has been linked to mast cells (MCs) in squamous cell carcinoma (SCC) and non-small cell lung cancer. However, its expression is rarely detected in normal adult tissues, making it a potential target for selective cancer therapy (Kapellos et al., 2013; Khan et al., 2017). Over expression of survivin leads to increased cell survival by inactivating Apaf-1, caspase-9 and Mdm2, which in turn suppresses p53, resulting in uncontrolled cell division due to Cdk1 activation and promotes tumor growth (Mobahat et al., 2014). Additionally, studies have revealed that survivin interacts with Second Mitochondria-derived Activator of Caspase/Direct Inhibitor of Apoptosis-binding Protein with Low pI (Smac/DIABLO), which acts as an antagonist to apoptotic inhibitors and aggregates survivin. This interaction breaks up the protein-protein interaction (PPI) between survivin and other proteins, which is difficult to achieve through small molecules (Mobahat et al., 2014; Pavlyukov et al., 2011).

*Nelumbo nucifera*, or the sacred lotus, possesses numerous biological and pharmacological properties, making it a plant of scientific interest. The seeds of lotus are also known as *Nelumbinis semen*. *In vitro* studies have reported the anticancer activity of *N. nucifera*. For example, Paudel and Panth (Paudel and Panth, 2015) established that the leaf extract of *N. nucifera* exhibited potent anticancer activity against melanoma, prostate, and gastric cancer cells owing to the presence of 7-hydroxydehydronuciferine compounds in the leaves (Liu et al., 2014). In another study, aporphine alkaloids in leaves were shown to be effective antioxidant and anticancer agents. In addition, Dasari et al. examined the anticancer effect of neferine, an alkaloid compound from lotus seeds (Dasari et al., 2020). Neferine showed strong anticancer activity, inducing apoptosis and autophagy as reported in their study. The results of the studies described above, *N. nucifera* has potential as an agent of clinically significant bioactive compounds.

Chemo-profiling of plant extracts through gas chromatography-mass spectrometry (GC-MS) is gaining increasing demand because of such multitude of applications for the analysis of herbals and botanicals of medicinal interest (Altameme et al., 2015). Docking enables the identification of novel compounds of therapeutic interest based upon compound’s binding affinity for the target protein or the compound-target interactions at a molecular level (Meng et al., 2011). The current study aimed to evaluate the phytoconstituents present in the methanolic extract of lotus seeds, which has been claimed to possess antitumor activity and induce apoptosis by functionally blocking survivin via molecular docking studies coupled with an *in vitro* study. The methanolic extract was used in the study as methanol has been found to be superior or more efficient in the extract of lower molecular weight polyphenols having anticancer or antioxidant potential. The methanol extract was found more active than other extracts. This may be attributed to the polar strength of methanol and it capability to extract more phenolic or polyphenolic compounds from the plant material. There are a plenty of literature (Paudel and Panth, 2015) that claim the superiority of methanolic extract over other extracts.

## 2 Materials and methods

### 2.1 Plant collection and authentication

The seeds of *N. nucifera* (lotus seeds) cultivar were selected for the study and were collected from the Gudlavalleru lake, Krishna District, Andhra Pradesh, India. They were authenticated by Dr. P. Srinivasa Rao, Assistant Professor, Department of Botany, P. B. Siddhartha College of Arts and Science (Autonomous), Vijayawada, Andhra Pradesh, India. A sample specimen was submitted to the Department to maintain a reference of the same and was marked as voucher specimen ID of PBS/BOT/004. The collection of plant material, its authentication and experiments conducted on the plant species comply with institutional and national guidelines.

### 2.2 Extraction of plant material

The seed material was washed with distilled water and dried under room temperature. It was finally grinded to obtain a powder using a blender. The powder sample was subjected to maceration with varying polarity solvents, namely, petroleum ether, *n*-hexane, acetone and methanol. The yields of four crude extracts were determined by the following formula. Following their extraction, the samples were kept at 4°C in a refrigerator for further analysis.

### 2.3 *In vitro* studies

The *in vitro* anticancer activities (anti-proliferative activity) of the extracts were carried out on ovarian cell lines.

#### 2.3.1 Cell culture

Two human ovarian cancer cell lines, namely, SKOV3 and A2780, were obtained from the National Centre for Cell Sciences (NCCS), Pune, India. These cells were cultured as a monolayer in McCoy’s 5A medium modified supplemented with 10% foetal bovine serum, 100 U/mL penicillin and 100 mg/mL streptomycin. The SKOV3-CisR and A2780-CisR cell lines were derived by culturing the original SKOV3 and A2780 cell lines, respectively, over 12 months with gradually increasing doses of cisplatin. The cells were enzymatically cultured for 2 minutes using a solution of 0.25% trypsin and 1 mM EDTA. Furthermore, 125 cm^2^ flasks of 75 cm^2^ plastic flasks were sub-cultured at a density of 2.2 × 104 cells/cm^2^. The culture medium was changed at 48 h intervals. The cell confluence was determined based on microscopic examination focusing on 80% confluence. The cells were then treated after being seeded for 12 h to prevent cell differentiation.

#### 2.3.2 MTT assay

In a 96-well tissue culture plate, SKOV3, A2780, SKOV3-CisR and A2780-CisR cells (100 µL per well) were placed. The cell count per well was 105 cells. Test samples of petroleum ether, n-hexane and acetone extracts were added to SKOV3 and A2780 ovarian cancer cells. The concentrations of the extracts ranged from 5 to 320 μg/mL (5, 10, 20, 40, 80, 160, and 320 μg/mL). The cells were seeded and incubated for 12 h, and then further incubated for 24, 48, and 72 h. The methanolic extract of lotus seeds (MELS) was tested against SKOV3, A2780, SKOV3-CisR, and A2780-CisR cells at concentrations ranging from 5 to 320 μg/mL (5, 10, 20, 40, 80, 160 and 320 μg/mL). The cells were seeded in triplicate and incubated for 12 h, followed by incubation for 24, 48, and 72 h. All test samples were prepared using a 20 µL amount of culture media. Injected 15 µL of MTT reagent each well, prepared in PBS medium, resulting in a final concentration of 0.5 mg/mL. The reagent volume was modified in accordance with the cell culture volume. The cells were cultured for the duration of 3 h at a temperature of 37°C until the presence of intracellular purple formazan crystals was observed using a microscope. To reach well 100 µL of DMSO was introduced. The mixture was gently agitated on an orbital shaker for 1 h at room temperature. The amount of DMSO was modified according to the volume of the cell culture. An absorbance plate reader was used to quantify the absorbance at OD570 nm for each well. The percentage of cell viability (Mosmann, 1983; Turan et al., 2017) was calculated as follows:

$$\text{Cell viability rate }\left(\%\right)=\frac{\left(\text{experimental}\;\text{group}\;\text{OD}-\text{zero}\;\text{adjustment}\;\text{group}\;\text{OD}\right)}{\left(\text{control}\;\text{group}\;\text{OD}-\text{zero}\;\text{adjustment}\;\text{group}\;\text{OD}\right)}\;X\;100$$

#### 2.3.3 Apoptosis assay

Apoptosis was assessed using FITC-labeled Annexin V/PI double labelling and flow cytometry analysis. In brief, SKOV-3, A2780 SKOV3-CisR, and A2780-CisR cells were exposed to the methanolic extract at the concentration that inhibits 50% of cell growth (IC_50_) for a duration of 24 h. Cells were collected and preserved at the specified period. Apoptosis was subsequently assessed using the FITC Annexin V Apoptosis Detection Kit II (BD Biosciences, Mississauga, ON) as per the instructions provided by the manufacturer. The BD Biosciences C6 flow cytometer was used to measure the proportions of cells in the early and late phases of apoptosis. The data were processed utilizing FlowJo 10.1 software. A minimum of 10,000 cells were enumerated for each measurement. The following controls were used to set up gates: unstained cells, cells with FITC Annexin V only, and cells with PI only (Demir et al., 2018).

#### 2.3.4 Cell cycle analysis

The cell cycle distribution of SKOV3-CisR and A2780-CisR cells after treatment with MELS was determined using flow cytometry. Before the experiment, the cells were adjusted to a density of 5 × 106 cells/mL and left to bind for 24 h before drug application. The cells were fixed after 24 h of exposure to MELS at IC_50_. The cell pellet was resuspended in 500 μL of PBS and fixed in 70% cold ethanol at −20°C for at least 2 h. After centrifugation at 1,000 rpm for 10 min followed by double rinsing with PBS, the cells were treated with 500 μL PI/RNase solution containing 400 μL propidium iodide and 100 μL ribonuclease A. The mixture was then incubated for 10 min at room temperature and PI/RNase mixture was added into it. The equipment selected for measuring the amount of DNA in cells was the BD Biosciences C6 flow cytometer, with an argon laser light source at 488 nm and a 630 nm band-pass filter. A total of 10,000 events were registered per sample and evaluated in terms of percentage relative to the untreated control population of the cells using a BD FACSDiva (BD Biosciences) (Li et al., 2019).

### 2.4 GC-MS analysis

GC-MS analysis was performed on a GC Clarus 500 Perkin Elmer system, which included an AOC-20i autosampler and a gas chromatograph interfaced with a mass spectrophotometer (GC-MS) instrument. The analysis used the following conditions: a column with an Elite-1 fused silica capillary column (30 × 0.25 mm ID x 1EM df, consisting of 100% dimethyl polysiloxane), operated in electron impact mode at 70 eV; helium (99.999%) was used as the carrier gas at a constant flow of 1 mL/min, and an injection volume of 0.5 EI was employed (split ratio of 10:1). The injector temperature was set at 250°C, and the ion-source temperature was set at 280°C. The oven temperature was programmed to start at 110°C (isothermal for 2 min), then increase by 10 ^°^C/min to 200°C, followed by a 5 ^°^C/min increase to 280°C, ending with a 9-min isothermal period at 280°C. Mass spectra were recorded at 70 eV, with a scan interval of 0.5 s and fragments ranging from 40 to 550 Da (Fitrianto et al., 2020; Shah et al., 2023).

### 2.5 *In silico* studies

#### 2.5.1 Molecular docking

The docking of GC-MS eluted phytocompounds to human survivin protein (PDB: 3UIH) was carried out using AutoDock Vina (Prasanth et al., 2023). The input files needed for running this program were prepared using AutoDock software. The AutoDock files were prepared by adding polar hydrogen atoms and Gasteiger charges. For X, Y and Z dimensions, the grid box size in AutoDock Vina was maintained at 15, and the binding center was x = −34.831; y = −8.98 and z = 3.038 (Killari et al., 2023). However, the energy range was maintained at eight, which was the default setting (DSNBK et al., 2023; Prasanth et al., 2021). The ligand-binding affinity was expressed as a negative score, with kcal/mol as a unit. Each ligand input generated nine ligand poses with different binding energies, as did the AutoDock Vina script. The pose with the highest binding affinity was extracted from the docked complex using an in-house Perl script. Using the Biovia Discovery Studio 2020 Visualizer, we studied ligand-protein interactions. The rationale for the selection of human survivin protein (3UIH) is that it a prominent anti-apoptotic protein which acts by directly binding and inhibiting caspase 3 activity.

#### 2.5.2 Drug-likeliness and ADMET analysis

The structures of phytochemical compounds were acquired in SDF format from PubChem. Subsequently, these compounds were subjected to drug-likeness predictions using the DruLiTo software (Ezugwu et al., 2024; Singh et al., 2024). Investigating the pharmacokinetic properties of the ligands is necessary to understand their roles in the body. To assess the ADMET profiles of the ligands, the Swiss ADME, admetSAR and ProTox-II open source web servers were employed (Singh et al., 2024; Archana et al., 2023).

## 3 Results and discussion

### 3.1 Extraction and percentage of yield

The lotus seeds were extracted using various solvents such as petroleum ether, n-hexane, acetone and methanol by maceration method. The yields of the petroleum ether extract (PELS), n-hexane extract (HELS), acetone extract (AELS), and methanol extract (MELS) were determined to be 3.44%, 2.85%, 5.98%, and 8.25% w/w, respectively.

### 3.2 MTT assay

Significant positive findings were obtained while testing the anti-proliferative effects of Nelumbinis semen extract on ovarian cancer cells (Meng et al., 2011; Shah et al., 2023). Various concentrations of MELS, AELS, PELS, NELS and cisplatin, ranging from 5 to 320 μg/mL, were incubated with SKOV3 and A2780 cells. The findings demonstrated that the extracts had cytotoxic action against ovarian cancer cell types. After 72 h of incubation, MELS, AELS exhibited dose dependent decreased cell viability to 2.4%, 45.3% respectively on SKOV3 cells, decreased cell viability to 5.3%, 46.4% respectively after 48h of incubation on A2780 cells. MELS also exhibited significant decrease cell viability to 9.7% after 72 h of incubation on SKOV3 resistance cells and decrease cell viability to 10.3% after 48 h of incubation on A2780 resistance cells. The Lotus seed extracts demonstrate anticancer action, as evidenced by their IC_50_ value, which is the concentration at which 50% of cellular proliferation is inhibited. The study found that MELS had a substantial effect on SKOV3, A2780, SKOV3-CisR, and A2780-CisR cells, with IC_50_ values of 79.73 ± 0.91, 100.18 ± 2.42, 115.87 ± 2.2, and 138.86 ± 2.46 μg/mL correspondingly, compared to other extracts (Supplementary Figure S1; Supplementary Table S1).

### 3.3 Apoptosis assay

**FIGURE 1:**
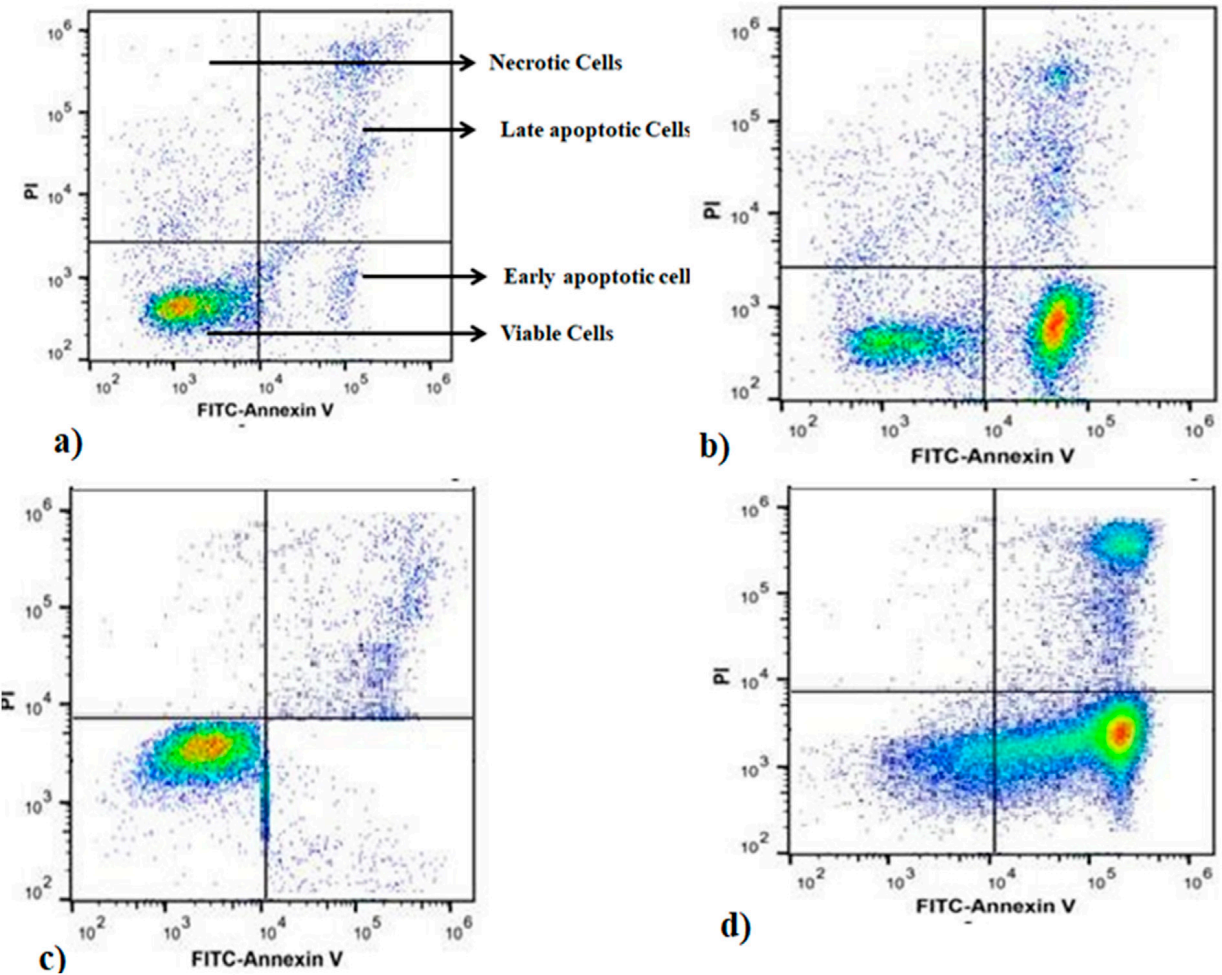
Annexin-V FITC/PI staining of apoptosis at 24 h of incubation. **(A)** SKOV3-CisR control, **(B)** A2780-CisR cells, **(C)** A2780-CisR control, **(D)** MELS treated SKOV3-CisR cells.

Apoptosis is a form of cell death. Disruption of the normal regulation of apoptosis results in the development of pathological states, such as cancer and autoimmune disorders. Consequently, scientists have concentrated their endeavours on devising strategies aimed at specifically triggering apoptosis in cancer cells (Baru Venkata et al., 2023). To assess apoptotic activity and quantify the rate of apoptosis, MELS was introduced to the cells at a dose that produces half of the maximum effect (EC_50_) for duration of 72 h. The cells were then examined using annexin V and propidium iodide (PI) staining. Annexin V was utilised to identify cells in the initial phases of apoptosis by detecting the externalised phosphatidylserine (PS) on the cell membrane, which is a distinctive alteration during apoptosis. Conversely, PI was employed to identify cells in the later stages of apoptosis and dead cells. PI is used to label cells that have a compromised cell membrane. Living cells do not exhibit binding to annexin V and PI, as indicated by the annexin-V−/PI− phenotype. Early apoptotic cells specifically bind to annexin V but not PI, resulting in the annexin-V+/PI− phenotype. Late apoptotic cells exhibit binding to both molecules, leading to the annexin-V+/PI + phenotype. Dead cells alone bind to PI, as indicated by the annexin-V−/PI + phenotype (Mahassni and Al-Reemi, 2013). After incubating the A2780-CisR cells with the MELS for 72 h, we observed that 78% of the cells in the treatment group tested positive for annexin V. In contrast, only 0.5% of the cells in the control group tested positive for annexin V. The treatment of SKOV3-CisR cells resulted in 82% of the cells exhibiting annexin V positivity, while the control group showed only 0.4% annexin V positivity compared to the control. The observations suggest that MELS has a substantial effect in triggering apoptosis in A2780-CisR and SKOV3-CisR cells (Figure 1).

### 3.4 Cell cycle analysis

**FIGURE 2:**
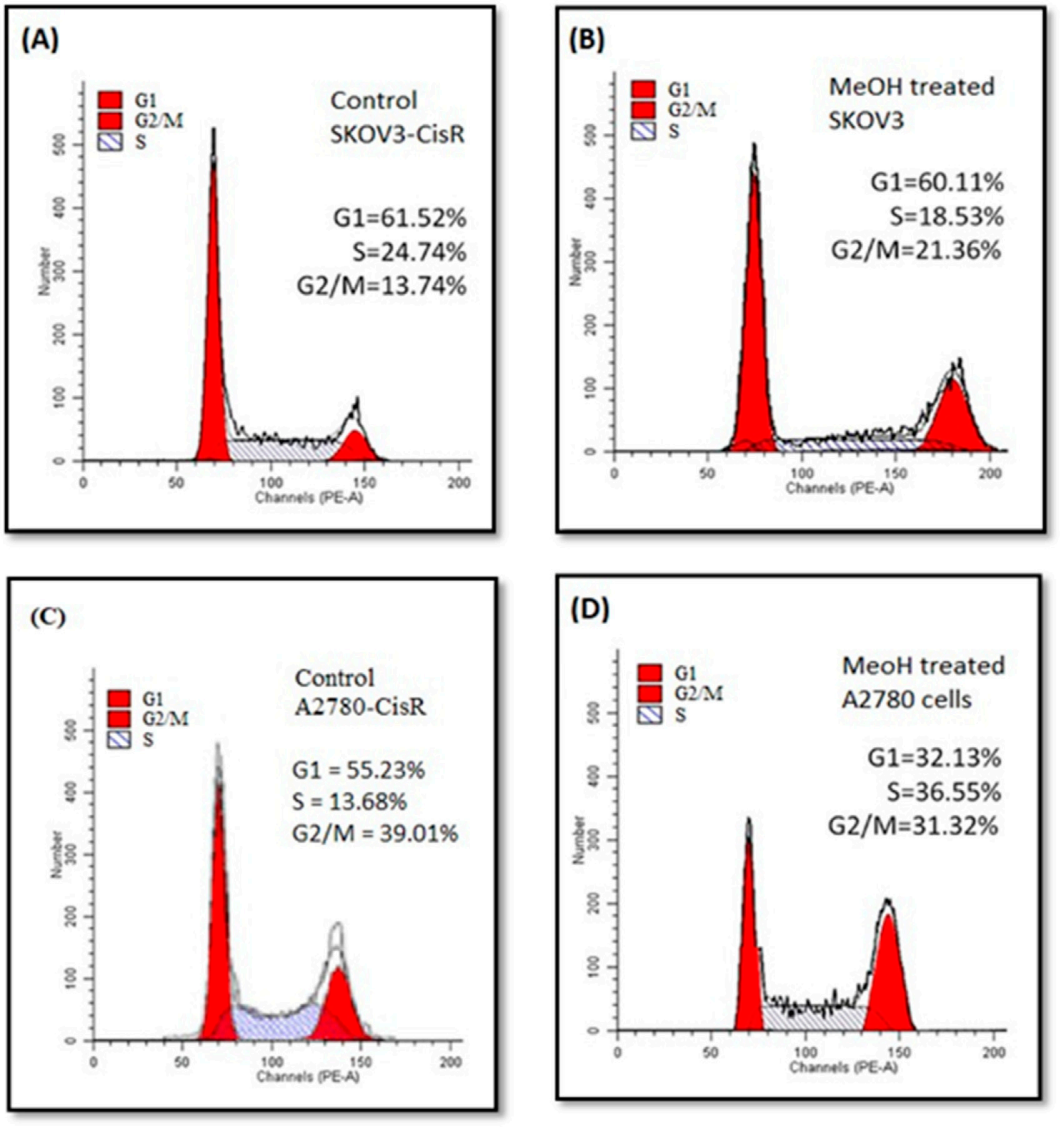
Flow cytometric cell cycle distribution analysis. **(A)** SKOV3-CisR control cells, **(B)** MELS treated SKOV3-CisR, **(C)** Control cells of A2780-CisR, **(D)** MELS treated A2780-CisR cells.

The results of cell cycle analysis using flow cytometry are presented in Figure 2. Treatment of SKOV3-CisR cells with MELS at a concentration of 100 μg/mL led to a significant reduction in the proportion of cells in G1 and S phases, from 61.52% to 60.11% and 24.75%–18.53%, respectively, compared to the control. Similarly, treatment of A2780-CisR cells with MELS resulted in a significant decrease in the proportion of cells in G1/S and G2/M phases, from 55.23% to 32.13% and 39.01%–31.32%, respectively, compared to the control. The cell cycle encompasses several checkpoints that enable the cell to repair its damaged DNA. Checkpoints at the G1/S and G2/M transitions play a crucial role in regulating cell cycle progression. However, the loss of these checkpoints prior to completing DNA repair can trigger the apoptotic cascade, leading to cell death (Hartwell and Kastan, 1994; Kastan and Bartek, 2004). Hence, it is clear that targeting the cell cycle will serve as an excellent source of novel anticancer chemicals (Carnero, 2002). After treatment with MELS, the cell-cycle distributions were found to be greatly aggregated at the G2/M phase. This indicates that MELS has the ability to cause cell-cycle arrest in ovarian cancer cells.

### 3.5 GC-MS analysis of MELS

The identified compounds in the MELS, as determined by GC-MS analysis, are listed in order of their column elution time (Supplementary Figure S2). A total of 14 compounds were detected (Table 1), accounting for 64.97% of the whole extract. Among the detected compounds, oleic acid (6.59%), stigmast-5-en-3-ol (5.64%), 2,3-dihydroxypropyl-cis-13-docosenoate (4.8%), lupeol (4.08%), and lupanol (4.06%) were the most dominant. The most representative compounds identified were lucenin 2 (3.91%), isocolchicine (3.11%), phytol (2.82%), β-amyrin (2.64%), 3,4,3′,4′-tetrahydrospirilloxanthin (2.63%), phytofluene (2.49%), oleanolic acid (2.37%), betulin (2.03%), glyceryl linolenate (1.91%), and 1-oxo-forskolin (1.83%).

**TABLE 1:** Phytoconstituents identified in MELS by GC-MS.

Name of phytoconstituent	R. Time	I. Time	F. Time	Area	Area%	Height	A/H
Lupeol	1.061	1.030	1.105	685259	3.43	168066	4.08
Carotene-1.1′,2.2′- tetrahydro-1.1′-dimethoxy	1.392	1.375	1.430	72449	0.36	27522	2.63
Isocolchicine	10.160	10.155	10.210	77835	0.39	25035	3.11
Lupanol	10.220	10.210	10.280	108983	0.55	26833	4.06
Stigmast-5-en-3-ol	10.505	10.440	10.540	162031	0.81	28753	5.64
Oleanolic acid	11.200	11.150	11.240	52566	0.26	22136	2.37
Glyceryl linolenate	16.728	16.695	16.770	98823	0.49	51686	1.91
Lucenin 2	17.256	17.055	17.525	2711916	13.57	694269	3.91
Betulin	18.606	18.545	18.640	191201	0.96	94199	2.03
1-Oxo-forskolin	18.672	18.640	18.715	221003	1.11	120602	1.83
Beta-amyrin	20.775	20.720	20.815	84646	0.42	32095	2.64
Phytofluene	20.967	20.935	21.055	229449	1.15	91980	2.49
Phytol	26.032	25.995	26.095	51785	0.26	18385	2.82
Oleic acid	27.470	27.465	27.610	43525	0.22	6,606	6.59

### 3.6 Molecular docking

Molecular docking is an essential tool in computer-aided drug design, which aids in explaining ligand’s binding affinity towards a protein target. It aids in identifying new therapeutic drugs and predicting ligand-protein interactions (Cetin, 2022; Cetin et al., 2023). It further enables structure-activity relationship studies and combinatorial library design to yield faster and more cost-effective drug discovery. The validation of molecular docking protocol was done by re-docking of the co-crystal ligand into the active site of receptor molecule (PDB: 3UIH) and confirming the conformation and orientation of a specific pose having a RMD value of not more than 2%. For protein-ligand docking, the active binding site (binding pocket) known as Smac/DIABLO of survivin was chosen for the docking study as it has pro-apoptotic function.

In the present study, MELS exhibited remarkable anticancer properties compared to other solvent extracts. GC-MS analysis was performed to discern the phytoconstituents responsible for the anticancer activity. Fourteen compounds were identified, and their docking score and interactions with survivin were evaluated through molecular docking study, as shown in Tables 2, 3. Based on the molecular docking studies, the binding affinity of ligands were oleic acid (−6.1 kcal/mol) > stigmast-5-en-3-ol (−5.9 kcal/mol) > phytol (−5.8 kcal/mol) > glyceryl linolenate (−5.6 kcal/mol).

**TABLE 2:** Results of molecular docking of 14 phytochemicals [with survivin (PDB: 3UIH)] identified in MELS by GC-MS.

Ligand	Binding energy (kcal/mol)
Oleic acid	−6.1
Stigmast-5-en-3-ol	−5.9
Phytol	−5.8
Glyceryl linolenate	−5.6
Lupeol	−4.9
Oleanolic acid	−4.9
Lupanol	−4.8
Phytofluene	−4.4
Beta amyrin	−4.1
Betulin	−3.8
Isocolchicine	−3.8
Lucenin 2	−3.7
1-Oxo-forskolin	−3.2
Carotene-1.1′,2.2′- tetrahydro-1.1′-dimethoxy	−3.2

**TABLE 3:** Binding energies and interaction details of top-scored phytochemicals with survivin protein (PDB: 3UIH).

Ligands	Protein	Binding affinity ΔG (kcal/mol)	Amino acids involved and distance (Å)	
			Hydrogen-bond interactions	Hydrophobic interactions
Oleic acid	Survivin (PDB: 3UIH)	−6.1	ASP A:71 (3.34), ASP A:72 (4.65), GLU A:76 (3.98)	LEU A:64 (4.15, 6.25), TRP A:67 (3.38), HIS A:80 (6.31, 6.56)
Stigmast-5-en-3-ol		−5.9	-	TRP A:67 (3.57), HIS A:80 (5.26)
Phytol		−5.8	ASP A:72 (3.48), GLU A:76 (3.49)	LEU A:64 (3.80), HIS A:80 (5.01)
Glyceryl linolenate		−5.6	LYS A:79 (5.45), GLU A:76 (4.48, 5.17)	GLU A:65 (6.36), GLY A:66 (4.55), ASP A:72 (4.13)

**FIGURE 3:**
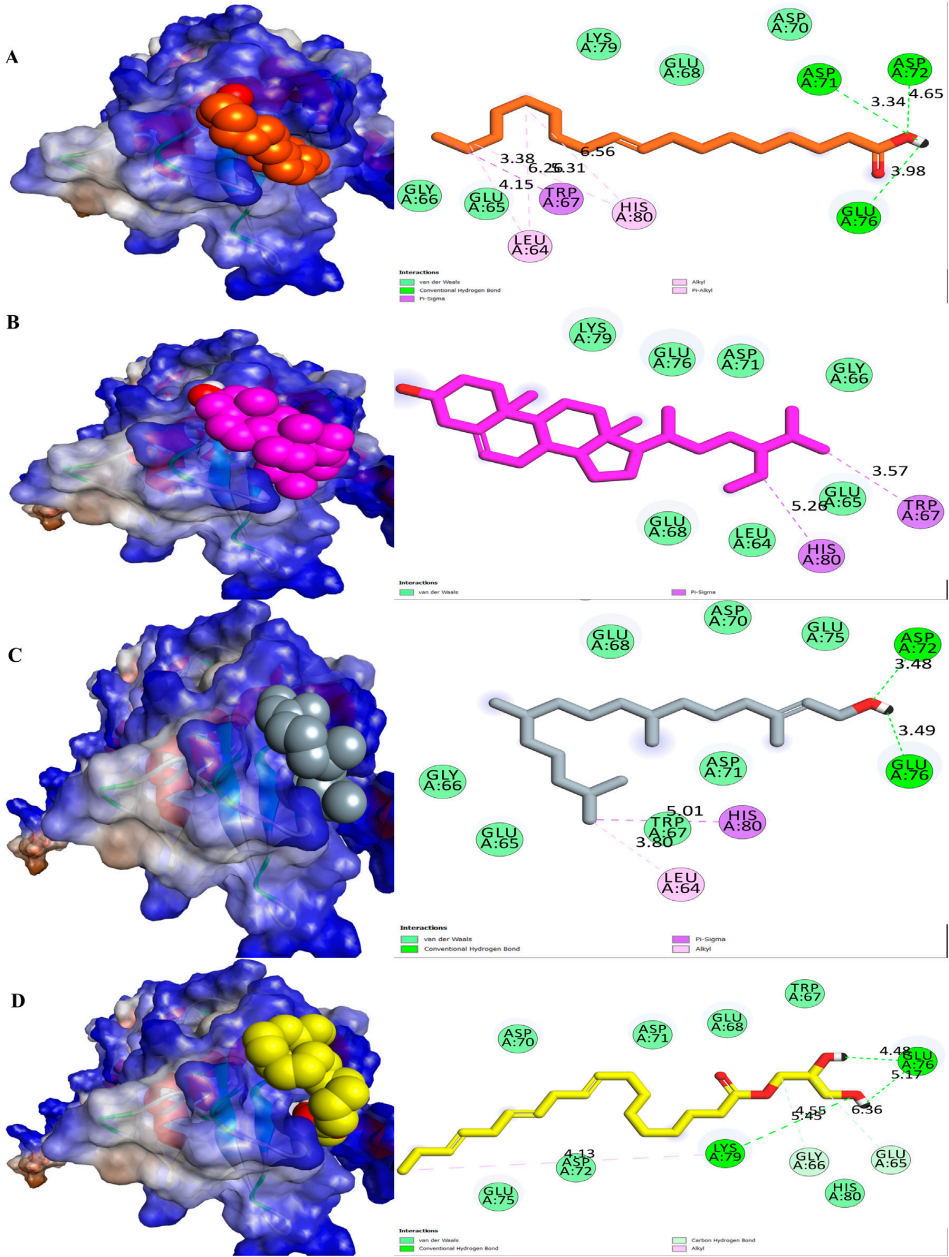
Molecular surface view and 2D representation of interactions between phytochemicals with the survivin. **(A)** Oleic acid, **(B)** Stigmast-5-en-3-ol, **(C)** Phytol and **(D)** Glyceryl linolenate.

Table 3 illustrates the binding affinities and interactions of various ligands with the survivin protein (PDB: 3UIH) (Figure 3). Among the ligands examined, oleic acid exhibited the strongest binding affinity with the survivin protein at −6.1 kcal/mol. Oleic acid formed hydrogen-bond interactions with ASP A:71, ASP A:72, and GLU A:76, while engaging in hydrophobic interactions with LEU A:64, TRP A:67, and HIS A:80. Stigmast-5-en-3-ol, with a binding affinity of −5.9 kcal/mol, interacted primarily through hydrophobic contacts with TRP A:67 and HIS A:80. Phytol, with a binding affinity of −5.8 kcal/mol, engaged in hydrogen-bond interactions with ASP A:72 and GLU A:76, while participating in hydrophobic interactions with LEU A:64 and HIS A:80. Glyceryl linolenate, with a binding affinity of −5.6 kcal/mol, formed hydrogen-bond interactions with LYS A:79, GLU A:76, and ASP A:72, and established hydrophobic contacts with GLU A:65, GLY A:66, and ASP A:72.

**FIGURE 4:**
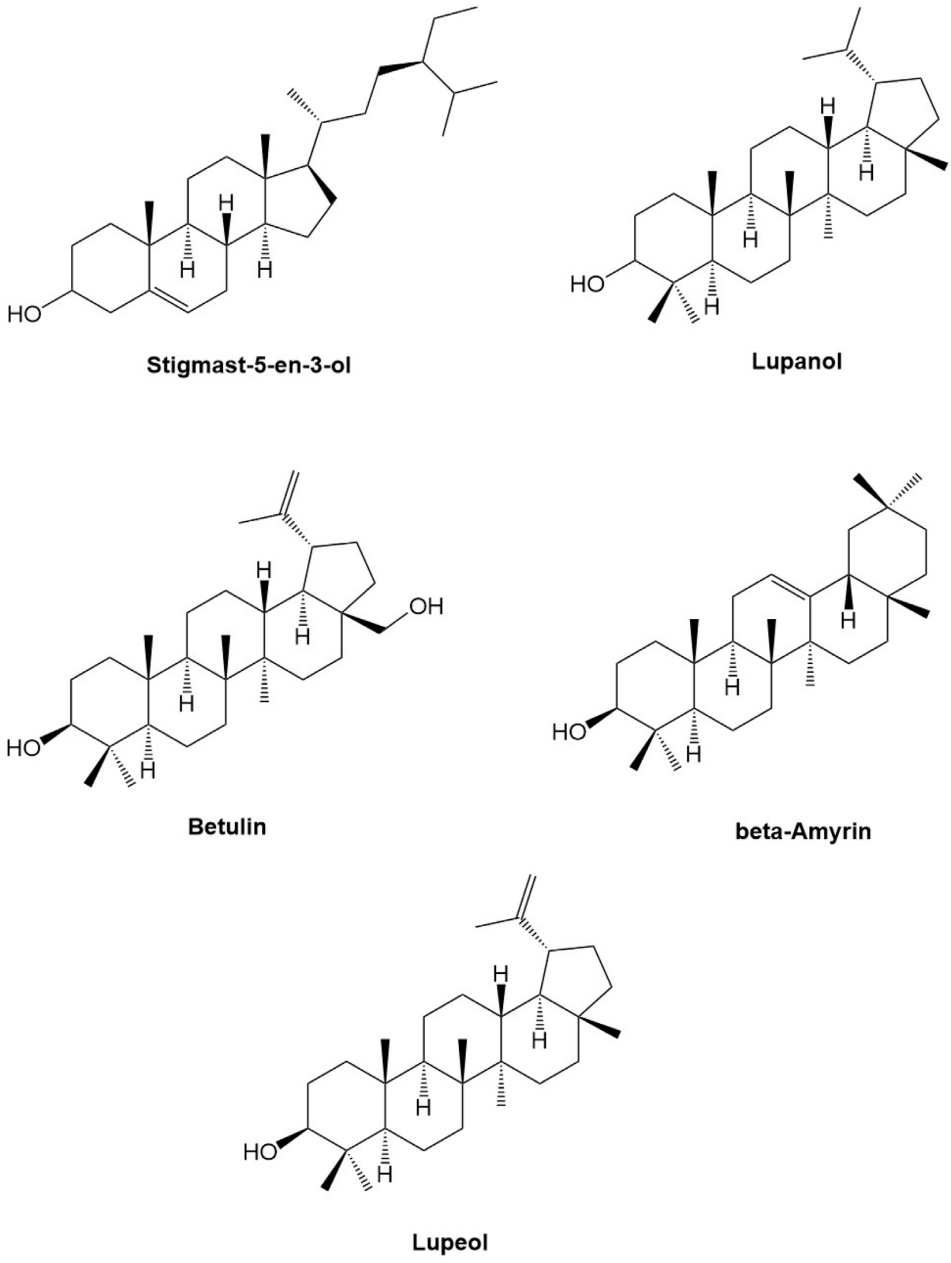
Lead scaffolds of some representative bioactive phytochemicals.

According to previous research conducted by Foroughi and colleagues (Singh et al., 2024), several phytoconstituents, including berberine, carvacrol, crocetin, crocin, curcumin, picrocrocin, piperine, and thymol, have been found to have stronger binding affinities with survivin. A SAR study of docking results demonstrate that the most bioactive phytochemicals (lead scaffolds of their structures are presented in Figure 4) inhibit survivin by binding with Asp71, Glu76, Glu65, Lys62, and Glu63 through hydrogen bond interactions. In our study, we also observed that these phytoconstituents, specifically stigmast-5-en-3-ol and phytol interact with Asp71, Asp72, Glu76, and Lys79 through hydrogen bond interactions, which confirms that they inhibit survivin by interacting with some of the aforementioned amino acids. A results of *in vitro* anticancer activity (IC_50_ values) concords the inhibitory potential (binding affinity, kcal/mol) observed in the docking study. Looking into the lead structural scaffolds one can be apparently believe that the steroidal framework or pentacyclic triterpenoid structural framework is essentially important for the anticancer potential of MELS along with the inhibition of human survivin protein. The cyclic hydrocarbon structure is involved in hydrophobic interactions and the hydroxyl groups participates in polar hydrogen bonding with the amino acid residues (catalytic active site) of surviving protein molecule as depicted in the preceding section.

The binding energy of oleic acid, a phytoconstituent, was found to be higher than that of other phytocompounds studied by Foroughi et al. (Singh et al., 2024) with a value of −6.1 kcal/mol. The anticancer properties of oleic acid have been demonstrated through its ability to inhibit the expression of HER2, a well-known oncogene that is involved in the development, progression, and spread of several human cancer. The previous study conducted by Fernando et al. demonstrated that stigmast-5-en-3-ol exhibited significant antiproliferative effects on HL-60 (leukemia) and MCF-7 (breast cancer) cell lines. Specifically, it was found to have IC_50_ values of 37.82 and 45.17 μg/mL for these cell lines, respectively (Fernando et al., 2018). As per the study conducted by the de Alencar et al. (2023), phytol has been demonstrated to exhibit potent anticancer properties, as evidenced by its ability to inhibit the growth of sarcoma −180 and human leukemia (HL-60) cells with IC_50_ values of 18.98 ± 3.79 and 1.17 ± 0.34 μM, respectively. The presence of oleic acid, stigmast-5-en-3-ol and phytol in MELS is likely responsible for its potent anticancer activity, as reported in previous studies. Our study presents a novel finding, as we report for the first time the inhibition of survivin with oleic acid, stigmast-5-en-3-ol, and phytol. To validate their potential as an anticancer agent through the inhibition of survivin, additional experimental research is necessary in the future.

### 3.7 Drug-likeliness

The results of the GC-MS analysis of MELS revealed the presence of 14 phytocompounds that were evaluated for drug-likeness using the DruLiTo method. These drug-like properties were assessed in accordance with Lipinski’s rule of five, which includes criteria such as logP ≤ 5, HBD ≤ 5, HBA ≤ 10, MW ≤ 500, TPSA ≤ 140, and AMR between 40 and 130 (Kumar Pasala et al., 2023). These parameters are important for considering a molecule as drug-like, as they affect its bioavailability, absorption, receptor-drug interactions, metabolism, and toxicity, which are all important factors for drug candidates to possess (Schneider, 2013). Additionally, the molecule size is also a crucial factor, especially for transmembrane transportation (Kumar Pasala et al., 2023). The study of drug likeness, based on the physicochemical nature of bioactive compounds, is an initial criterion for judging drug likeness. Lipinski’s rule of five provides a structural similarity between an idealistic/rationalized drug synthetic structure and a bioactive compound. However, it is important to note that a drug candidate does not necessarily need to follow all the rules to be considered a potential drug candidate. Previous studies have shown that the lack of oral bioavailability does not necessarily affect the activity or pharmacokinetic potencies of a drug (Bickerton et al., 2012). From this analysis, two compounds, isocolchicine, and 1-oxo-forskolin, were identified as ideal molecules for further examination as they obey Lipinski’s rule of five. However, it is important to note that the majority of natural products have been found to deviate from the Lipinski rule of five, as evidenced by various studies (James et al., 2023; Gandhi et al., 2022). The results of physicochemical properties are listed in Supplementary Table S2.

### 3.8 ADMET analysis

ADMET is a process that assesses the pharmacokinetic properties of a drug and its potential for clinical use. One of the advantages of ADMET analysis is that it can help identify issues in the early stages of drug development, which can reduce the likelihood of clinical trial failures (Schneider, 2013). This study examined for three lead compounds, with solubility, intestinal absorption, dermal permeability, and Caco2 permeability being key parameters (Dahlgren and Lennernäs, 2019).

Supplementary Table S3 provides a detailed summary of the phytoconstituents and their pharmacokinetic properties, which were obtained using different computational tools, *viz.*, SwissADME, admetTSAR, and ProTox-II. The compounds identified through molecular docking, including oleic acid, stigmast-5-en-3-ol, phyol and glyceryl linolenate, showed moderate to poor solubility, which resulted in low absorption in the gastrointestinal tract. However, according to the ProTox-II studies, all four of these compounds were found to be safe, and none of them exhibited hepatotoxicity, carcinogenicity, mutagenicity, or cytotoxicity. In terms of LD_50_, oleic acid falls in Class 4 with a dose of 480 mg/kg, stigmast-5-en-3-ol belongs to Class 4 with a dose of 890 mg/kg, phytol falls in Class 5 with a dose of 5,000 mg/kg and glyceryl linolenate is in Class 6 with a dose of 39800 mg/kg. Based on this information, it can be concluded that all of these compounds are safe for human use.

## 4 Conclusion

The methanolic extract of lotus seeds (MELS) possesses anti-proliferative effects against ovarian cancer cells, possibly by inducing cell death and hindering the cell cycle. *In vitro* study demonstrated significant inhibition of SKOV3 (IC50: 79.73 ± 0.91), A2780 (IC50: 100.18 ± 2.42), SKOV3-CisR (IC50: 115.87 ± 2.2) and A2780-CisR (IC50: 138.86 ± 2.46) cells by MELS, compared to acetone, petroleum ether, n-hexane extracts, and the standard drug, cisplatin. Furthermore, MELS resulted in a substantial increase in apoptosis cell count to 78% in A2780-CisR cells and 82% in SKOV3-CisR cells, whereas a significant reduction in the G1 and G2/M phases of cells treated with MELS when compared to the control group. To identify the potential phytocompounds present in the MELS, we conducted GC-MS analysis, which led to the identification of 14 phytochemical compounds. Molecular docking analysis revealed that oleic acid, stigmast-5-en-3-ol, phytol and glyceryl linolenate exhibited remarkable binding affinities of -6.1, -5.9, -5.8 and -5.6 kcal/mol, respectively against human survivin. Our findings suggest that phytochemicals present in MELS might have potential anticancer effect against ovarian cancer, and further investigation is required in order to fully elucidate the mechanisms of anticancer action.

## Data Availability

The original contributions presented in the study are included in the article/Supplementary Material, further inquiries can be directed to the corresponding authors.
