# Supplementary material for: Lotus seed (Nelumbinis semen) extract: anticancer potential and chemoprofiling by in vitro, in silico and GC-MS studies
Source: Front Chem. 2024 Dec 13;12:1505272. doi: 10.3389/fchem.2024.1505272 (PMC11671802; doi:10.3389/fchem.2024.1505272)
Supplement: Supplementary file 1 [file DataSheet1.docx]

Supplementary Material

# Supplementary Figures and Tables

## Supplementary Figures

**
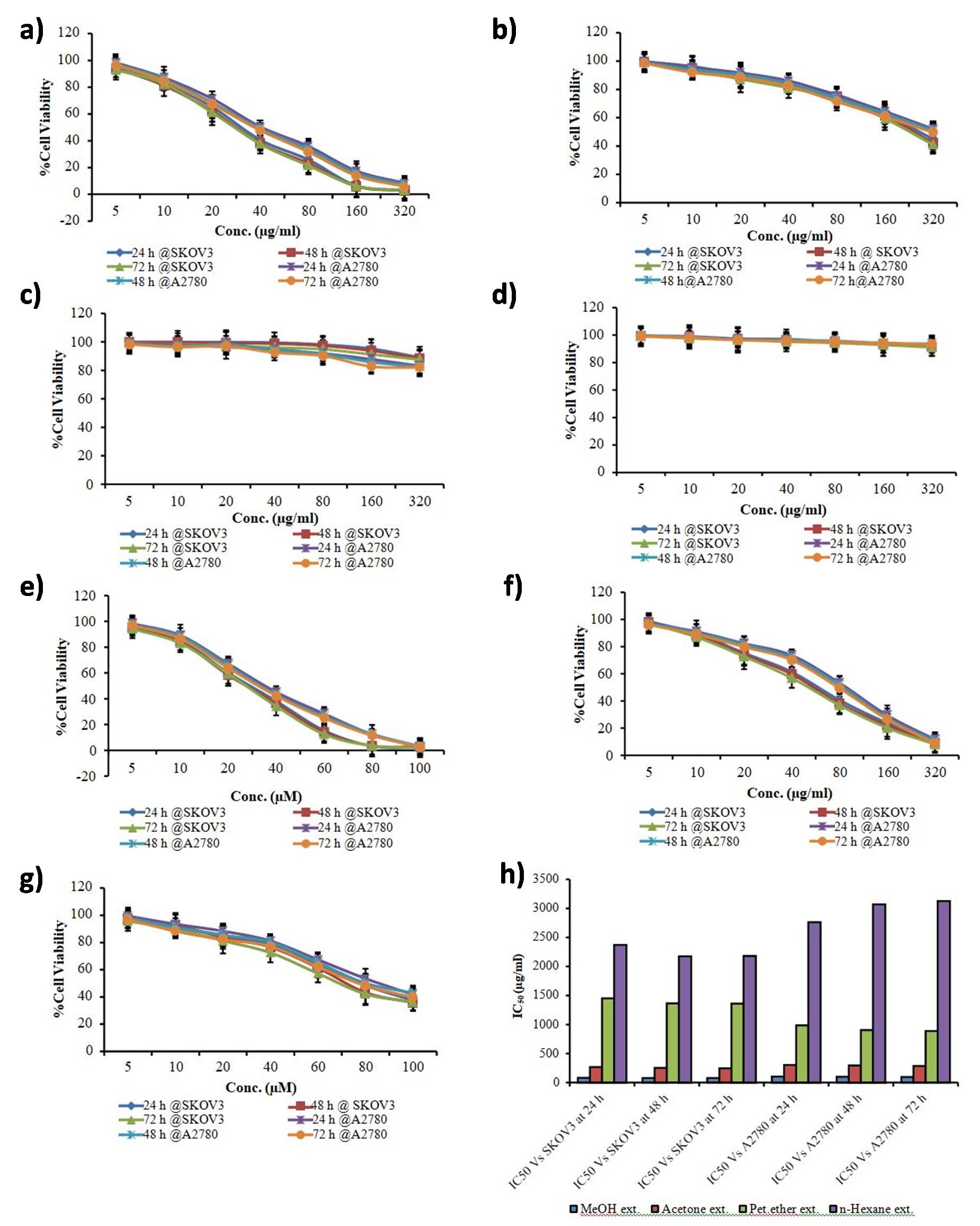
**

**Supplementary Figure 1** Results of MTT assay. **(**a) MeOH extract, (b) Acetone extract, (c) Pet. ether extract, (d) *n*-Hexane extract, (e) Cisplatin, (f) MeOH extract against resistant cells, (g) Cisplatin against resistant cells, (h) IC_50_ values of extracts against cell lines.

**Supplementary Figure 2** GC-MS chromatogram representing the separated bioactive phytoconstituents of MELS.

## Supplementary Tables

**Supplementary Table 1** IC_50_ of various extracts of lotus seed against cell lines.

| **Table 1a** Effect of extracts on SKOV3 | | | | |  | **Table 1b** Effect of extract on A2780 | | | | | | |
| --- | --- | --- | --- | --- | --- | --- | --- | --- | --- | --- | --- | --- |
| **Extract** | **IC_50_** | | | **Mean± SEM** |  | **Extract** | **IC_50_** | | | | | **Mean± SEM** |
| MELS | 81.53 | 78.55 | 79.12 | 79.73±0.91 |  | MELS | 104.42 | 100.06 | | 96.05 | | 100.18±2.42 |
| AELS | 266.53 | 254.2 | 246.68 | 255.8±5.7 |  | AELS | 302.98 | 294.82 | | 285.61 | | 302.98 ±5.02 |
| PELS | 1450.7 | 1363.1 | 1361.8 | 1391±29.4 |  | PELS | 986.38 | 903.27 | | 888.7 | | 926.12±30.42 |
| NELS | 2372.1 | 2174.2 | 2180.7 | 2242±64.91 |  | NELS | 2762.75 | 3068.5 | | 3125.2 | | 2985.48±112.5 |
| Cisplatin | 39.4 | 39.42 | 39.13 | 39.13±0.09 |  | Cisplatin | 44.81 | 44.45 | | 43.46 | | 44.24±0.40 |
|  |  |  |  |  |  |  |  |  | |  | |  |
| **Table 1c** Effect of extracts on SKOV3-CisR | | | | |  | **Table 1d** Effect of extracts on A2780-CisR | | | | | | |
| **Extract** | **IC_50_** | | | **Mean± SEM** |  | **Extract** | **IC_50_** | | | | **Mean± SEM** | |
| MELS | 119.75 | 116.01 | 111.85 | 115.87±2.2 |  | MELS | 143.29 | 138.63 | 134.67 | | 138.86± 2.46 | |
| AELS | - | - | - |  |  | AELS | - | - | - | |  | |
| PELS | - | - | - |  |  | PELS | - | - | - | |  | |
| NELS | - | - | - |  |  | NELS | - | - | - | |  | |
| Cisplatin | 81.74 | 77.67 | 76.54 | 78.65± 1.58 |  | Cisplatin | 87.85 | 87.17 | 83.13 | | 86.05±1.47 | |

**Supplementary Table 2** Physicochemical properties of phytoconstituents identified in MELS.

| **Compound** | **MW** | **logp** | **Alogp** | **HBA** | **HBD** | **TPSA** | **AMR** | **nRB** | **Violates Lipinski’s Rule** |
| --- | --- | --- | --- | --- | --- | --- | --- | --- | --- |
| Oleanolic acid | 412.02 | 9.052 | 2.13 | 3 | 0 | 17.07 | 134.03 | 1 | Yes |
| Carotene-1,1',2,2'- tetrahydro-1,1'-dimethoxy | 535.99 | 13.408 | 8.969 | 2 | 0 | 18.46 | 205.82 | 20 | Yes |
| Isocolchicine | 373.97 | 1.575 | -1.241 | 7 | 0 | 71.06 | 112.92 | 6 | No |
| Lupanol | 375.99 | 12.717 | 2.378 | 1 | 0 | 0 | 129.11 | 1 | Yes |
| Lucenin 2 | 585.97 | -2.204 | -5.65 | 16 | 0 | 44.76 | 146.48 | 5 | Yes |
| Phytol | 319.97 | 11.084 | 4.242 | 1 | 0 | 9.23 | 105.47 | 15 | Yes |
| Oleic acid | 311.97 | 10.129 | -0.04 | 2 | 0 | 26.3 | 85.33 | 17 | Yes |
| Stigmast-5-en-3-ol | 370.04 | 11.595 | 1.3 | 1 | 0 | 0 | 123.88 | 6 | Yes |
| Betulin | 398.04 | 9.714 | 2.141 | 2 | 0 | 0 | 131.81 | 2 | Yes |
| 1-Oxo-forskolin | 375.96 | 0.823 | 0.135 | 7 | 0 | 69.67 | 103.96 | 3 | No |
| Beta amyrin | 439.97 | 13.681 | 5.472 | 1 | 0 | 9.23 | 148.71 | 2 | Yes |
| Phytofluene | 480 | 14.712 | 11.123 | 0 | 0 | 0 | 195.03 | 19 | Yes |
| Lupeol | 382.04 | 11.901 | 3.231 | 1 | 0 | 0 | 130.04 | 1 | Yes |
| Glyceryl linolenate | 315.98 | 6.478 | -0.897 | 4 | 0 | 26.3 | 97.3 | 17 | Yes |

**Supplementary** **Table 3** ADMET properties of phytochemicals of MELS.

| **Phytocompounds** | **SwissADME** | | | | | | | | **admetSAR** | | | | | | | | **ProTox-II** | | | |
| --- | --- | --- | --- | --- | --- | --- | --- | --- | --- | --- | --- | --- | --- | --- | --- | --- | --- | --- | --- | --- |
|  | **log P o/w** | **Water Solubility** | **GI Absorption** | **Lipinski’s Rule** | **Veber’s Rule** | **PAINS Alert** | **TPSA** | **Lead Likeliness** | **HIA** | **BBB** | **Caco-2** | **CYP1A2** | **CYP2C19** | **CYP2C9** | **CYP2D6** | **LD_50_ (mg/kg)** | **Hepatotoxicity** | **Carcinogenicity** | **Mutagenicity** | **Cytotoxicity** |
| Oleanolic acid | 3.68 | Poorly soluble | Low | Yes | Yes | 0 | 57.53 | No | 1 | 0.776 | 0.8353 | 0.9169 | 0.9025 | 0.8258 | 0.9485 | 2000 (Class 4) | Active | Active | Inactive | Inactive |
| Carotene-1,1',2,2'- tetrahydro-1,1'-dimethoxy | 12.64 | Poorly soluble | Low | No | No | 0 | 18.46 | No | 0.99 | 0.955 | 0.7256 | 0.8132 | 0.7739 | 0.8902 | 0.9332 | 3200 (Class 5) | Inactive | Inactive | Inactive | Inactive |
| Isocolchicine | 3.09 | Soluble | High | Yes | Yes | 0 | 8309 | No | 0.986 | 0.787 | 0.5119 | 0.9045 | 0.9025 | 0.9071 | 0.9231 | 19 (Class 2) | Inactive | Inactive | Inactive | Active |
| Lupanol | 4.75 | Poorly soluble | Low | Yes | Yes | 0 | 20.23 | No | 1 | 0.984 | 0.8248 | 0.6166 | 0.8298 | 0.7576 | 0.9712 | 500 (Class 4) | Inactive | Inactive | Inactive | Inactive |
| Lucenin 2 | 1.12 | Soluble | Low | No | No | 1 | 291.4 | No | 0.916 | 0.687 | 0.9096 | 0.8801 | 0.9102 | 0.8119 | 0.9445 | 1213 (Class 4) | Inactive | Inactive | Active | Inactive |
| Phytol | 4.85 | Moderately Soluble | Low | Yes | No | 0 | 20.23 | No | 0.985 | 0.938 | 0.6445 | 0.9046 | 0.791 | 0.9071 | 0.8278 | 5000 (Class 5) | Inactive | Inactive | Inactive | Inactive |
| Oleic acid | 4.01 | Moderately Soluble | High | Yes | No | 0 | 37.3 | No | 0.995 | 0.954 | 0.8371 | 0.9107 | 0.9467 | 0.8972 | 0.8954 | 480 (Class 4) | Inactive | Inactive | Inactive | Inactive |
| Stigmast-5-en-3-ol | 5.05 | Poorly soluble | Low | Yes | Yes | 0 | 20.23 | No | 1 | 0.974 | 0.7953 | 0.9291 | 0.9025 | 0.9125 | 0.8977 | 890 (Class 4) | Inactive | Inactive | Inactive | Inactive |
| Betulin | 4.47 | Poorly soluble | Low | Yes | Yes | 0 | 40.46 | No | 0.997 | 0.923 | 0.8252 | 0.9045 | 0.9026 | 0.9071 | 0.9071 | 2000 (Class 4) | Inactive | Inactive | Inactive | Inactive |
| 1-Oxo-forskolin | 2.55 | Soluble | High | Yes | Yes | 0 | 110.1 | No | 0.736 | 0.785 | 0.5663 | 0.7797 | 0.8748 | 0.8892 | 0.8799 | 2550 (Class 5) | Inactive | Inactive | Inactive | Inactive |
| Beta amyrin | 4.63 | Poorly soluble | Low | Yes | Yes | 0 | 20.23 | No | 1 | 0.962 | 0.831 | 0.8575 | 0.6636 | 0.803 | 0.8597 | 7000 (Class 6) | Inactive | Inactive | Inactive | Inactive |
| Phytofluene | 13.61 | Poorly soluble | Low | No | No | 0 | 189.1 | No | 0.99 | 0.944 | 0.6999 | 0.7354 | 0.9168 | 0.9099 | 0.9491 | 5700 (Class 6) | Inactive | Inactive | Inactive | Inactive |
| Lupeol | 4.69 | Poorly soluble | Low | Yes | Yes | 0 | 20.23 | No | 0.997 | 0.959 | 0.8499 | 0.8619 | 0.732 | 0.8184 | 0.9047 | 2000 (Class 4) | Inactive | Inactive | Inactive | Inactive |
| Glyceryl linolenate | 4.47 | Poorly soluble | Low | No | No | 0 | 66.76 | No | 0.935 | 0.523 | 0.6453 | 0.7257 | 0.89 | 0.88 | 0.8518 | 39800 (Class 6) | Inactive | Inactive | Inactive | Inactive |
